# Biocontrol of *Meloidogyne* spp. in *Solanum lycopersicum* using a dual combination of *Bacillus* strains

**DOI:** 10.3389/fpls.2022.1077062

**Published:** 2023-01-04

**Authors:** Fernando Evaristo Díaz-Manzano, Deisy X. Amora, Ángela Martínez-Gómez, Lars Moelbak, Carolina Escobar

**Affiliations:** ^1^ Área de Fisiología Vegetal, Facultad de Ciencias Ambientales y Bioquímica, Universidad de Castilla-La Mancha, Toledo, Spain; ^2^ Chr Hansen A/S, AP Innovation Department, Hørsholm, Denmark

**Keywords:** *Bacillus paralicheniformis*, *Bacillus subtilis*, biological control, confocal, giant cells, rhizobacteria, root-knot nematodes, tomato

## Abstract

Root-knot nematodes (RKNs, *Meloidogyne* spp.) are obligate plant parasites that constitute a significant pest for agriculture worldwide. They penetrate the plant roots, reducing the uptake of water and nutrients, causing a significant impact on crop yield. One alternative on focus now for nematode management is biological control. Rhizobacteria within the *Bacillus* genus show multiple modes of action against plant-parasitic nematodes (PPNs) that can act alone or in combination. In this context, we evaluated a dual-strain bacteria combination (*B. paralicheniformi* FMCH001 and *B. subtilis* FMCH002) to reduce nematode infection in tomato plants. We evaluated mortality of larvae from Meloidogyne javanica *in vitro*, as well as eggs hatching after the treatment. Atraction, penetration, establishment, and reproduction assays *in vitro* or in pots in tomato plants infected with M. javanica and treated/ untreated with the dual-strain bacteria combination were also performed. Additionally, morphometric parameters comparing giant cells size from galls of treated and untreated plants by using confocal microscopy were also measured. The results showed that this combination of strains has nematicidal properties in the pre-infection phase by decreasing the egg-hatching, juvenile survival, and attractiveness to the roots. Furthermore, nematode establishment, gall formation, and, remarkably, giant cell development was severely impaired after the bacterial treatment, suggesting interference with morphogenetic mechanisms induced by the nematode during GCs development within the plant. Nematode reproduction in tomato plants was reduced independently of the application mode in soil, before or after bacterial treatment. The dual-strain combination was also effective against other PPNs (i.e. *Pratylenchus* spp.) and in different crops (soybean). Therefore, combining *B. paralicheniformis FMCH001* and *B. subtilis FMCH002* is an efficient agent for the biological control of *Meloidogyne* spp. by interfering with different stages of the nematode cycle as a result of multiple modes of action.

## Introduction

RKNs (*Meloidogyne* spp.) are one of the most important groups of nematodes due to their broad host range and the level of damage caused after their establishment in the root tissues of their hosts (Jones et al., 2013), causing agricultural losses of around $157 billion per year (Mokrini et al., 2018). They establish a sophisticated relationship within the host plants where they induce a local swelling called gall with the aid of specific effectors. Within the galls, they also trigger the formation of specialized nourishing cells called giant cells (Escobar et al., 2015). The intensive cropping with susceptible varieties, lack of awareness of farmers, and global warming have a significant impact on nematode disease aggravation and their emergence in new areas. Yet, nematode control is difficult due to its broad host range, soil-hidden nature, lack of resistant varieties, and the ban of many chemical nematicides because of their high environmental toxicity. Hence, there is an increasing demand for sustainable and efficient nematode-management strategies. These methods should be combined in the integrated pest management (IPM) system (Riyaz et al., 2022), which consists of combining different tactics, including host resistance (when available), rotation with nonhosts, sanitation, destruction of residual crop roots, biological control, among others.

Increasing the knowledge of IPM systems, including the development of new biotechnological tools against PPNs, is nowadays on focus (Alemu, 2020). In this respect, biological control using the soil rhizobacteria of the genus *Bacillus* is an alternative. They have shown the ability to produce antibacterial and antifungal secondary metabolites and to promote plant growth (Oliveira et al., 2014; Cao et al., 2019). Therefore, they drawn significant attention in recent years because of their safety for the environment and ability to suppress nematode populations in the soil (Engelbrecht et al., 2018; Topalović et al., 2020). Yet, the ability of different strains of the genus *Bacillus* spp. for PPNs control, reside in diverse effects. Some can be opportunistic, and feed from the nematodes, producing also proteases that affects and finally kill the nematode, they can increase plant tolerance due to its plant growth promoting effect (reviewed in Ahmad et al., 2021), or secrete toxic proteins as Cry5 that kill juveniles of *M. incognita* (Geng et al., 2017).

Rhizobacteria displays different methods of action to quell PPNs (Migunova and Sasanelli, 2021). The mechanism of nematode suppression can be categorized mainly in two major ways: a direct effect on the pre-infective juveniles, and an indirect effect on the post-infective juveniles and adults’ phase by triggering an immune response in the plant, delaying nematode development and reproduction. The direct effect involves production of enzymes (Lian et al., 2007; Corrales-Ramírez et al., 2017), antibiotics (Nadeem et al., 2021), volatile organic compounds and soluble secondary metabolites that are delivered around the rhizosphere (Oliveira et al., 2014). Some species of *Bacillus* spp. protects plants against nematodes through their high potential to form a biofilm on the roots which is a physical barrier to the nematode’s penetration. They can also form multi-species biofilms increasing their efficacy by combining the different strain-specific functions and diversity in the soil (Ansari and Ahmad, 2019). On the other hand, indirect mechanisms happen through interference with nematode behavior, by altering the root diffusates and affecting its recognition by the nematodes or altering the nematode feeding site development or sex female/male ratio inside the root tissue, or even by promoting plant growth (Mhatre et al., 2019). So far, some secondary metabolites can also trigger an immune reaction in the host plants, which leads to systemic resistance (Choudhary and Johri, 2009; Adam et al., 2014), rendering the plant less susceptible to pathogen infection. Some examples also point to enhanced beneficial effects combining both the plant growth attributes and their effect on nematodes control after co-inoculation of different rhizobacteria species (Ansari and Ahmad, 2019; El-Nagdi and Abd-El-Khair, 2019; Topalović et al., 2020).

Plant growth promoting rhizobacteria (PGPR; Lim and Kim, 2009) facilitate plant development by producing phytohormones (auxins, cytokines, ethylene, abscisic acid, etc.; Poveda and González-Andrés, 2021) that promote plant growth, helping to decrease disease severity, as the plants show more tolerance to the nematodes (Backer et al., 2018; Mhatre et al., 2019). Moreover, the phytohormones produced by rhizobacteria promote cell division and cell elongation (Khan et al., 2009; Arora et al., 2013), particularly, microbial auxins hormones increase the lateral and adventitious rooting leading to improved mineral and nutrient uptake (Arora et al., 2013), increasing nitrogen fixing and phosphate solubilization in the soil (Saeid et al., 2018; Jain et al., 2021). In this way, several studies demonstrated that inoculation of auxins producing PGPR in the field by seed application resulted in enhanced plant growth and nematicidal activity (Ruanpanun et al., 2010; Khan et al., 2012; Gamalero and Glick, 2020).

In this context, we use and evaluated a commercial blend of the dual-strain combination of *B. paralicheniformis* FMCH001 (previously identified as *B. licheniformis*) and *B. subtilis* FMCH002 on reducing nematode infection in plants. This blend is currently available as bionematicides in the market for managing different nematode species (Machado, 2022). We show that this specific dual-combination has nematicidal properties against *Meloidogyne* spp., interfering with egg hatching, juvenile penetration, female development, and reproduction during the nematode - *Solanum lycopersicum* interaction. The interference in the different stages of the nematode cycle is a result of the multiple modes of action of this *Bacillus* dual-strain bacteria combination that could effectively control RKNs in tomato.

## Material and methods

### Maintenance and extraction of *Meloidogyne* spp. inoculum

For the reproduction of *Meloidogyne javanica* Treub (1885; identified by morphology, isozymes pattern and specific primers by PCR described in Robertson et al. (2009)), *Cucumis sativus* (L.) cv. Hoffmanns Giganta seeds (Buzzy Seeds, Catalog Number: 02186) were surface sterilized, grown *in vitro*, inoculated with *M. javanica* J2, and maintained as described in Díaz-Manzano et al. (2016). *Meloidogyne incognita* (source: collection NPPO_NL E1318; identified by morphology and isozymes pattern) population was increased and maintained in *Solanum lycopersicum* cv. Tiny Tim (Moles Seeds) grown under greenhouse conditions. The nematode eggs were extracted from egg masses with 0.5% sodium hypochlorite, rinsed with water, and collected on a 25-μm sieve. For soil experiments, the nematode eggs were placed in a Baermann funnel, and the hatched second-stage juveniles (J2) were collected every 24 hours and stored in the fridge until the inoculation of the experiments. For *in vitro* assays in plates, J2s were obtained after hatching the egg masses grown in monoaxenic cultures in sterile conditions in tap water at 25°C following Díaz-Manzano et al. (2016).

### Bacteria treatment

Spores of *B. paralicheniformis* FMCH001 and *B. subtilis* FMCH002 were obtained through fermentation, spray-dried, and formulated as a wettable powder. The formulation contained 3,2 x 10^11^ spores of each of the strains per gram of wettable powder. For the treatments of roots and seeds, the powder was resuspended in tap water at the desired concentration and applied as drenching or seed coating, respectively. *Bacillus paralicheniformis* FMCH001 and *Bacillus subtilis* FMCH002 have been originally isolated from the soil environment, were classified through genomic analysis and the strains are part of Chr. Hansen Culture Collection.

### Hatching and mortality assay

#### Formulated spores

Spore suspensions at different concentrations were tested for the ability to cause mortality and impair the hatching of *Meloidogyne* spp. 100 J2 per replica (for the mortality assay) or eggs (for the hatching assay) in a 900µl bacterial suspension at the following concentrations: 3,2 x 10E+08, 1,6 x 10E+08, 3,2 x 10E+07, 3,2 x 10E+06were assayed in multiwell plates and incubated under gentle shaking in the darkness at 26°C for 96 hours (mortality assay) and ten days (hatching assay). At the end of the two incubations, mortality and hatching ratios were evaluated under a binocular. Four biological replicates (400 eggs or J2s) per treatment and experiment were tested in three independent experiments. To distinguish dead J2 from the immobile nematodes, 1 µL of 1N NaOH was added per well and their mobility was monitored after two minutes (Xiang and Lawrence, 2016). A blank formulation was prepared in the same concentration as the formulation with the bacteria to observe the chemical effect of the formulation products.

#### Supernatant free of bacterial cells

The nematicidal potential of metabolites released by the vegetative cells of the strains during growth was assessed in an *in vitro* mortality assay. Overnight cultures were used to inoculate 50 mL of MSgg (biofilm-promoting minimal medium) liquid to a starting OD600 of 0,05. The two strains were grown at 30°C, 200 RPM, for 24h and 48 h. The medium was centrifuged for 10 minutes at 4200 xg and then passed through 0,45 µm pore size membrane filters to collect the supernatant free of bacterial cells, at the end of each growth period. 100 surface-sterilized J2 were added to 400 µl of each bacteria supernatant in 50 µl of water suspension in a 24-well plate. Water and mock MSgg were used as control. The mortality ratio was measured after 48 hours of plate incubation at 26°C. 1 µL of 1N NaOH was added to each well to distinguish between dead and immobile juveniles and they were monitored after two minutes (Xiang and Lawrence, 2016).

### Nematode’s attraction and penetration experiments

Tomato seeds (*Solanum lycopersicum*) were surface sterilized (30% commercial bleach + 0,02% Triton X-100) for 15 min and washed three times with sterile water. Seeds were placed in a Petri dish on a wet filter paper, stratified at 4°C in darkness for 24 h, and then transferred to a growth chamber (26°C) for five days. The roots of the seedlings were submerged in a suspension of 1 x 10 ^6^ CFU/ml of *B. paralicheniformis* FMCH001 and *B. subtilis* FMCH002 combined. Water was used as a control. The seedlings were transferred to 120 mm square plates (five seedlings per plate) containing 20 ml of Pluronic gel 23%. The plates were placed in a vertical position in a growth chamber at 26°C. After 24 hours, 600 juveniles were distributed in five spots at the bottom edge of the plate. The plates were returned to the growth chamber and the number of nematodes around each root tip was recorded at 5 and 24 hours after the nematode inoculation (**Figure S3**). The roots at 24 hours and five days after nematode inoculation, were removed from the plate and stained with acid-fuchsin to assess penetration by counting the nematodes inside the root (Bybd et al., 1983). Root colonization of bacteria was confirmed by staining root samples with the nucleic acid-specific dye SYBR^®^ green (diluted 1:1,000; **Figure S4**). Samples were incubated at room temperature for five minutes and taken under the microscope for visualization.

### Tomato *in vitro* infection test

Seeds of *Solanum lycopersicum* (L.) cv. Roma VF (Fitó Seeds, Catalog. Number: 81045) were coated with the dual-strain combination of *B. paralicheniformis* FMCH001 and *B. subtilis* FMCH002 (1g of formulated product/Kg of seed). Uncoated seeds were used as control. Both coated and uncoated seeds were sown in 0.3% Gamborg medium (Gamborg et al., 1968). Seeds were arranged in 120 mm square plates (VWR^®^ Gosselin; España, Llinars del Vallès), 15 seeds in a row, followed by a light pulse for two hours to promote germination. Plates were individually wrapped in aluminum foil and grown vertically in a growth chamber at 26°C ± 1°C. 4 days after germination (dag) when roots were on average 2-3 cm long, every plantlet was inoculated with 10-15 J2 of *M. javanica* per root tip as described in Portillo et al. (2013). Plates were wrapped again in aluminum foil and evaluated 4 days post inoculation (gall scoring). A minimum of 140 seeds per treatment (uncoated and coated) were analyzed in three independent experiments.

### Greenhouse experiments

#### Tomato vs. *Meloidogyne*

The efficacy of the dual-strain combination was evaluated at two different timepoints: before and after nematode inoculation. Three weeks old tomato seedlings (*Solanum lycopersicum*) var. Roma, grown in vermiculite, were transferred to 1L pots containing 800 g of silver sand as substrate. One week after transplanting, seedlings were treated as follows: treatment 1 (drenched with dual-strain bacteria combination (day 1) + inoculated with *M. javanica* J2s (day 3)); treatment 2 (inoculated with *M. javanica* J2s (day 1) + drenched with dual-strain bacteria combination (day 3)); treatment 3 (inoculated with *M. javanica* J2s (day 1) + drenched with tap water (day 3)). Plants were fertilized fortnightly with NPK 3g/L and watered at plant demand (every ± 3 days). Plants were inoculated with 400 juveniles/pot of *M. javanica* or with 20mL/pot of dual-strain (10^8^ spores/ml) following the treatments indicated before. At the end of each experiment, the roots and aerial part of the tomatoes were harvested separately and weighed. Eggs were extracted from the tomato plants 8 weeks after inoculation based on the method of Hussey and Barker (1973) and 3 aliquots were counted under a stereo microscope to assess the total number of eggs per treatment.

#### Soybean vs. *Meloidogyne*

The efficacy of the bacteria treatment by seed coating on nematode control was evaluated with *Meloidogyne* spp., under greenhouse experiments. Soybean seeds variety Abelina coated with the dual-strain combination as described before, were sown in a 3-liter pot filled with sandy soil. By the sowing time, each seed was inoculated with 3000 eggs of *M. incognita*. Plants were grown in a greenhouse for 30 (one cycle) and 60 days (two cycles) at 26°C and at the end of each phase of the experiment, the eggs were extracted from the roots as described before.

#### Soybean vs. *Pratylenchus*

The seed coating was also evaluated for the control of the lesion nematode *Pratylenchus neglectus* in greenhouse experiments, using field soil naturally infested with the nematode. Initial nematode population was around 500 specimens/Kg. The nematode specie was identified by morphology at specialized diagnostic laboratory (ILVO Plant). To determine the nematode densities, nematodes were extracted from the soil using modified Cobb’s sieving and decanting, followed by the sugar flotation method from 100 g samples (Ingham, 1994). One soybean seed was sown in per pot and the experiment was kept in the greenhouse for 40 days at 26°C. At the end of the experiment, the roots were harvested, weighed, and taken to nematode extraction. Nematodes were extracted following the method proposed by Coolen and D'herde (1972). All the infectious forms were counted under an optical microscope.

### Confocal morphological analysis

14dpi (days post inoculation) galls from *in vitro* grown plants as previously described were hand-dissected, fixed with 3% glutaraldehyde overnight, sequentially dehydrated, and incubated overnight in BABB for clarification of the tissues following Cabrera et al. (2018). Galls were observed in auto-fluorescence and transmittance light with an Ar/Kr laser at 488 nm (Leica TCS SP8 laser scanning confocal microscope; Leica, Wetzlar, Germany). A spectral detector was set up at 505–585 nm for the green auto-fluorescence emission generated by glutaraldehyde. Leica Confocal Software LCS was used for image capture.

### Data processing

Data obtained were represented with histograms with mean values and/or percentages per line/treatment and standard errors ( ± SE). Statistical analyses were performed using Student’s t-test in the SPSS package (IBM, Armonk, NY, USA) and confidence intervals were established with a significance of 5% (P < 0.05; asterisk).

## Results

### The mortality, attraction, and penetration of J2, as well as eggs hatching, is severely affected after bacterial treatment

**Figure 1:**
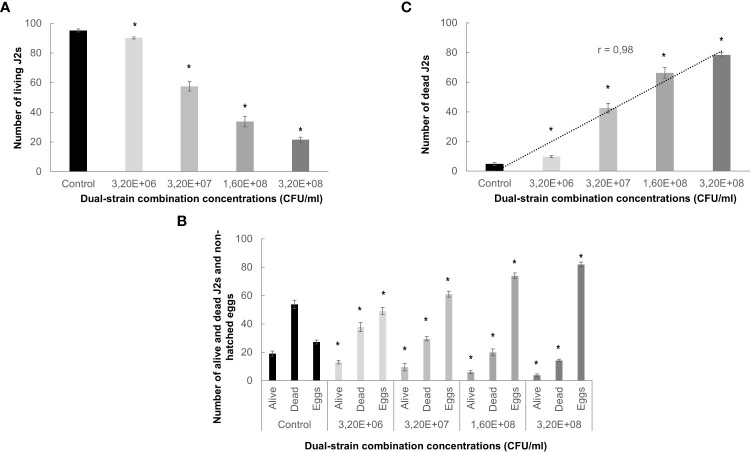
Effect of bacterial treatments on the mortality and hatching of *Meloidogyne javanica*. **(A)** Number of living J2s nematodes relative to the control for each treatment is represented in the Y axis. Nematodes were monitored after 96 hours. **(B)** Number of alive and dead J2s, and non-hatched eggs respect to the control for each treatment are represented in the Y axis. Eggs hatching was counted 10 days after incubation. 10mM magnesium sulfate was used as control. Four independent replicates of 100 eggs or J2s each were tested per treatment in three independent experiments. Dilutions from 1x of dual-strain bacteria combination (*B. paralicheniformis* FMCH001 and *B subtilis* FMCH002, 3,20E+08 CFU/ml), are indicated in the X axis. 1x is 3,20E+08 CFU/ml; 1/2x is 1,60E+08 CFU/ml; 1/10x is 3,20E+07 CFU/ml; 1/100x is 3,20E+06 CFU/ml. **(C)** Pearson correlation coefficient (r) between J2s mortality (y-axis) and the bacteria dilutions (x-axis) was 0,98, p<0.003. Bars represent mean ± SE. *Asterisks, significant differences respect to control (t-test; P<0.05). CFU is considered as the number of spores.

This study aims to explore and characterize the effectiveness of a combination of bacteria as a biological control method for *Meloidogyne* spp. We firstly analyzed its bionematicidal potential by assessing the effect of the dual-strain bacteria combination on the mortality and hatching of the nematodes. The treatment with the dual-strain bacteria showed a significant (p<0.05) effect on the mortality of the J2 (**Figure 1A**). Even in the more diluted treatment (1/100 from the stock, 3,2 10E +08 CFU/ml), a significant reduction of the percentage of alive nematodes as compared to the control was observed (25%), decreasing to 80% at 1x; **Figure 1A**). Additionally, we studied the hatching capacity of eggs of the same nematode species after the bacteria treatment, determining a significant (p<0.05) effect after incubation in all the dual-strain bacteria concentrations tested as compared to the control (**Figure 1B**). The undiluted bacteria showed only 20% of hatched eggs (**Figure 1B**). Interestingly, the presence of dead J2 correlated directly to the dilution of the bacteria spores (Pearson test: 0,980; p<0,003; **Figure 1C**). Additionally, we tested whether the formulation used for the dual-strain bacteria could have any effect on the J2 survival and eggs hatching. The results indicate that the formulation used for the dual-strain bacteria applied as 1x and 1/2x dilution did not show any significant effect on the nematodes’ mortality or hatching (**Figure S1A**), however, the two dilutions used for the dual-strain bacteria reproduced the significant reduction in both parameters shown in **Figure 1**.

**Figure 2:**
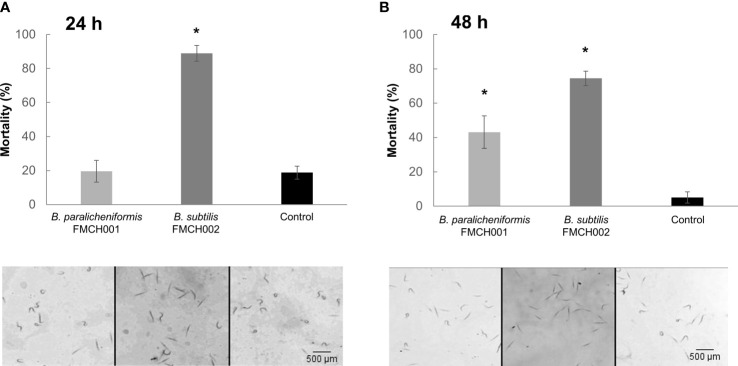
Effect of bacteria supernatants free of bacterial cells on the mortality of *Meloidogyne incognita*. Percentage of dead nematodes treated with supernatant of bacteria *B paralicheniformis* FMCH001; *B subtilis* FMCH002 as indicated, grown in MSgg for **(A)** 24 h and **(B)** 48 h. In the panels below, representative images of the nematodes shape in each of the bacteria treatments and the control. Curled nematodes are alive, straight nematodes, dead. Nematodes were monitored after 24 hours. MSgg media was used as control. 100 J2s were tested per treatment with three biological replicates. Bars represent mean ± SE. *Asterisks, significant differences respect to control (t-test; p < 0.05). Scale bar: 500µm.

The effect of each bacteria supernatant was evaluated on the mortality of J2s from *M. incognita* in an *in vitro* experiment. The results showed significant differences in the mortality rate of the J2 treated with the 24 hours growth supernatant of the bacteria strain *B. subtilis* FMCH001, but no significant effect with that of the bacteria strain *B. paralicheniformis* FMC002 compared to the mock treated J2 (**Figure 2A**). However, a significant (p<0.05) effect on the mortality of J2 was observed for both strains, *B. subtilis* and *B. paralicheniformis*, for the 48 hours growth supernatant (**Figure 2B**). The most pronounced effect was observed with the strain FMCH002, which showed severe mortality (≥70%; p<0.05; **Figure 2B**).

**Figure 3:**
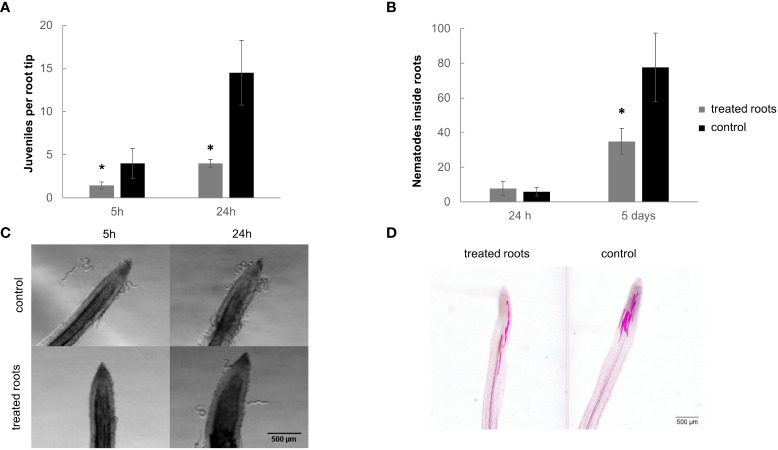
*M. incognita* juvenile attraction and penetration in tomato roots treated with the dual bacteria combination. **(A)** Number of juveniles around root tip at 5 hai and 24 hai. **(B)** Number of nematodes inside the roots at 24 hai and 5 dai. **(C)** A representative image of a tomato root tip surrounded by J2 from *M. incognita*. **(D)** A representative image of J2 after penetration on a tomato root tip. 600 juveniles were inoculated per plate. Roots were submerged in a suspension of 1 x 10E+06 CFU/ml of the dual-strain bacteria combination of *B paralicheniformis* FMCH001 and *B subtilis* FMCH002. Bars represent mean ± SE. *Asterisks, significant differences respect to control (t-test; p<0.05). Scale bar: 500µm. hai=hours after inoculation, dai=days after inoculation.

We also studied the effect of the dual-strain bacteria treatment on the plant-nematode attraction under *in vitro* conditions (**Figure 3**; **S3**). Significant differences (p<0.05) were encountered in the attraction experiments of *M. incognita* J2 in tomato plants root treated with the dual-strain combination of B. *paralicheniformis* FMCH001 and *B. subtilis* FMCH002 as compared to untreated roots five and 24h after inoculation (**Figures 3A, C**). The number of J2 around the roots was 3.5-fold reduced after 24 hours respect to the control (**Figures 3A, C**). These results are also in line with the differences shown in the number of J2 that penetrated the roots five days after inoculation (**Figures 3B, D**). Bacteria were observed on the surface of the roots (**Figure S4**) which indicates that the bacteria can colonize the roots after treatment.

### Nematode establishment, as well as galls/giant cells development, are compromised in tomato after bacteria treatment *in vitro*

**Figure 4:**
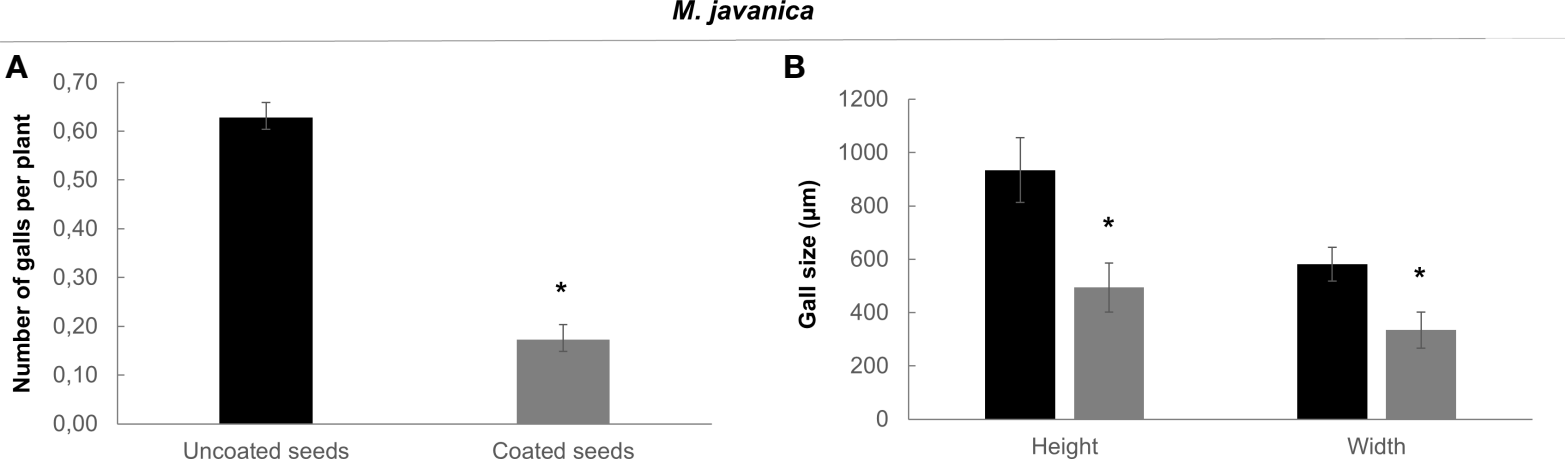
Effect of bacterial treatment (coated seeds) in the infection of *Solanum lycopersicum* (L.) cv. Roma VF with *M. javanica*. **(A)** *In vitro* grown tomato plantlets were evaluated at 4 days post inoculation. Bars represent number of galls per plant ± SE. 140 plants per treatment were tested in three independent experiments. **(B)** Effect of bacterial treatment in the galls size. Tomato galls were hand-dissected and evaluated 14 days post inoculation. Bars represent mean of galls size (height and width) ± SE for ten galls per treatment from three independent experiments. Seeds were uncoated (black) or coated (grey) with the dual-strain bacteria combination of *B paralicheniformis* FMCH001 and *B subtilis* FMCH002. *Asterisks represent significant differences respect to uncoated seeds (t-test; P<0.05). Black, uncoated seeds, grey, coated seeds.

**Figure 5:**
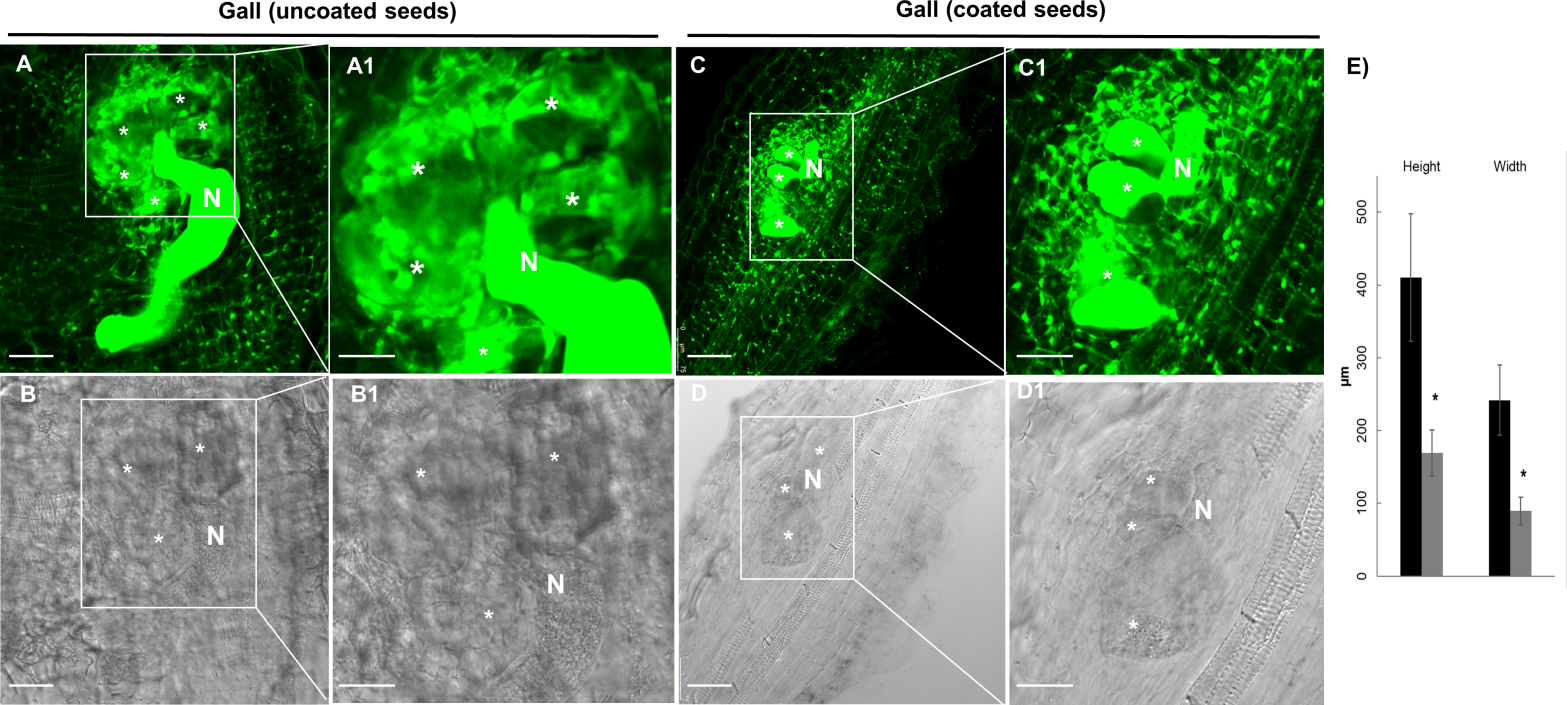
Representative confocal images of tomato, *Solanum lycopersicum* (L.) cv. Roma VF, root-galls infected with *M. javanica* at 14 dpi from bacteria-treated (coated seeds) and non-treated, control plants. **(A)** Auto-fluorescence of the glutaraldehyde (green), and **(B)** Transmission images of control (untreated) gall from tomato at 14dpi, **(C)** Fluorescence of the glutaraldehyde (green), and **(D)** Transmission images of galls from bacteria treated plants with seeds coating. **A1**, **B1**, **C1**, **D1**), close up images of the giant cells (GCs) and the nematode as indicated, to facilitate their observation. The GCs size showed differences between those treated with the dual-strain bacteria combination and tomato control galls;*, giant cells; N, nematode; Scale bars: 75µm. **(E)** Quantification of the GCs size. Bars represent mean of giant cells size (height and width) ± SE for fifty giant cells per treatment, from three independent experiments. *Asterisks represent significant differences respect to uncoated seeds (t-test; P<0.05). Seeds were uncoated (black) or coated (grey) with the dual-strain bacteria combination of *B paralicheniformis* FMCH001 and *B subtilis* FMCH002.

**Figure 6:**
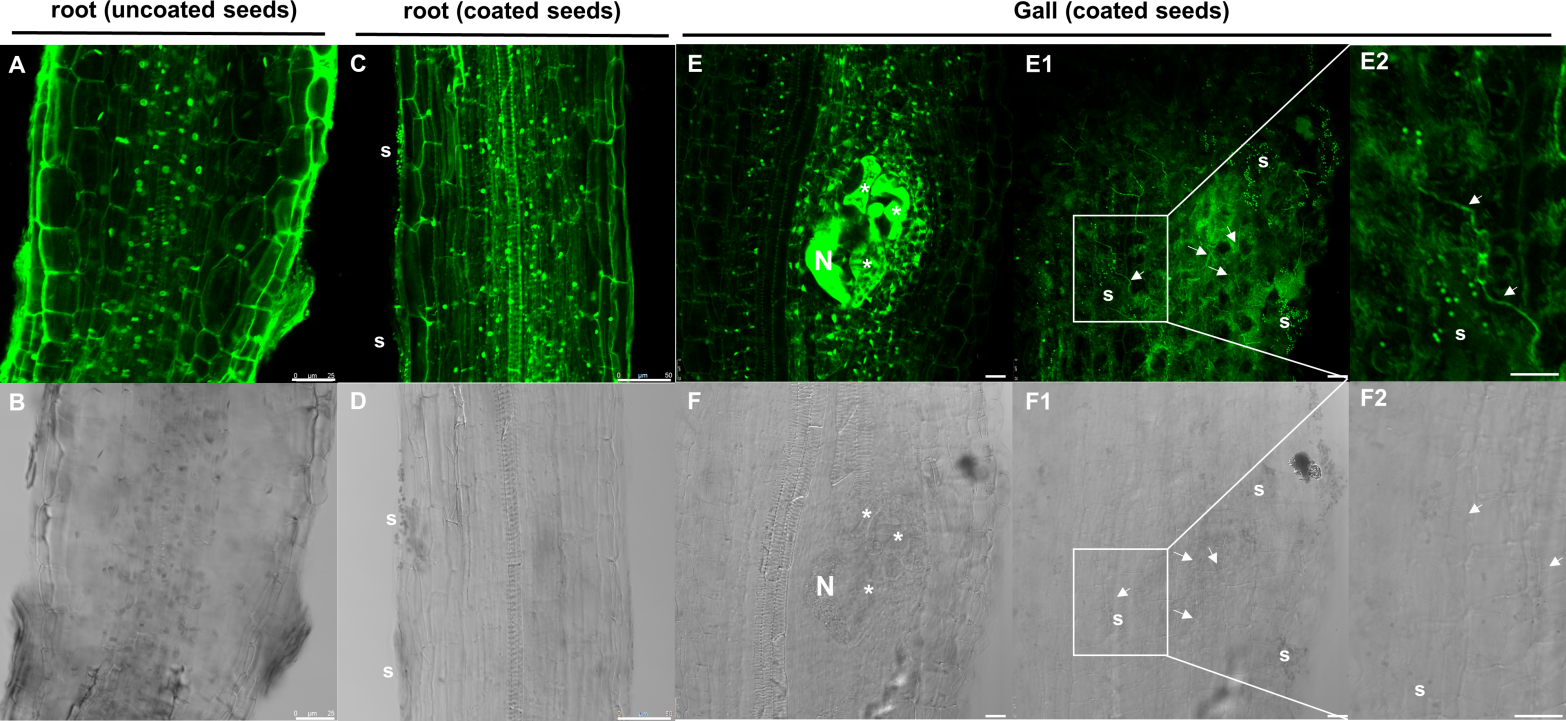
Representative images of auto-fluorescence (in green) and transmittance light (in grey) of bacteria in the tomato root external tissue and inside the galls. **(A, B)**, tomato uncoated root control. **(C, D)** tomato root coated with dual-strain bacteria combination. **(E, F)**, 14dpi gall of tomato root coated with dual-strain bacteria combination. **E1**, **F1**) upper part of a z-stack of the 14dpi gall of tomato root coated with dual-strain bacteria combination shown in **E, F**, **E2**, **F2**) close up images of E1 and F1, respectively. Data showed that tomato roots and galls/giant cells coated with the dual-strain bacteria combination of *B paralicheniformis* FMCH001 and *B subtilis* FMCH002 were colonized by spores (s) and/or vegetative cells. Asterisks, giant cells; N, nematode; white arrows, vegetative cells; s, spores. Scale bars: 25µm for **A, B, E, E1, E2, F, F1** and **F2**. 50µm for **(C)** and **(D)** Seeds were uncoated (black) or coated (grey) with the dual-strain bacteria combination of *B paralicheniformis* FMCH001 and *B subtilis* FMCH002.

We performed nematode infection tests *in vitro* in tomato plantlets grown from coated and uncoated seeds eight days post germination (dag). The number of galls per plant in the bacteria treated plants was severely reduced respect to the control (more than 72%; p<0.05; **Figure 4A**), therefore, seed coating significantly inhibited nematode establishment. We further analyzed whether the development of the galls was also inhibited by the bacterial treatment. The samples were fixed with glutaraldehyde and clarified with BABB to favor laser penetration and therefore the observation of the structure of the gall (see Materials and Methods). The fluorescence of the glutaraldehyde was used to image the whole galls by confocal microscopy where the internal structures of the galls are distinguished (**Figure 5**). The preservation of the galls and giant cells structures allowed us to measure their width and height to quantify any change in their size due to the treatment. The data showed that there are morphological differences in the development of the galls with the bacterial treatment, as those treated with bacteria showed significant differences in size (height and width) compared to the galls formed in the untreated plantlets, with a reduction around 57% for height and 53% for width (**Figure 4B**). In accordance, differences were also observed in the size of the giant cells with a significant (p<0.05) reduction of around 59% and 63% (height and width; respectively; **Figures 5A, C, A1, C1, B, B1; D, D1; E**) in the giant cells formed in plantlets from bacteria-coated seeds respect to the untreated. We could also detect spores and some filamentous structures from the bacteria in the gall’s tissues (**Figures 6E, E1, E2**), which confirms together with the data presented in **Figure S4** and (**Figures 6C, D**), that the bacteria can colonize not only control root tissues but also the infected tissues, galls, formed by the nematodes.

### RKNs reproduction rates are inhibited by the dual-strain bacteria treatment in tomato

**Figure 7:**
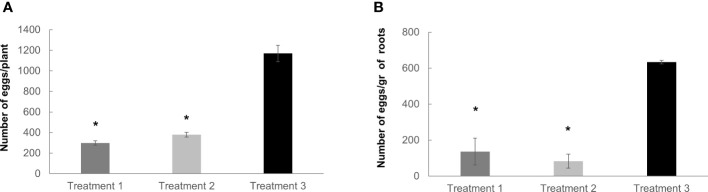
Nematode reproduction analysis after dual-strain bacteria treatments in tomato plants. The number of eggs obtained in tomato (*Solanum lycopersicum*) var. Rome inoculated with 400 J2/plant of *M. javanica* was quantified in all cases, **(A)** per plant, and **(B)** per gram of roots weight. Treatment 1 (irrigated with dual-strain bacteria combination, day 1, and inoculated with *M. javanica*, day 3); treatment 2 (inoculated with *M. javanica.* day 1, and irrigated with dual-strain bacteria combination, day 3) and treatment 3 (inoculated with *M. javanica*, day 1, and irrigated with only tap water, day 3). Treatment 3 was used as control of nematode infection. Number of eggs were scored eight weeks post inoculation (from day 1). Bars represent mean ± SE, n=24 plants tested per treatment in three independent experiments. Concentration of 1x dual-strain bacteria combination of *B paralicheniformis* FMCH001 and *B subtilis* FMCH002, 3,2 x 10E+08 CFU/ml. Statistics are always comparisons to treatment 3 as a control. *Asterisks represent significant differences (t-test; P < 0.05).

Reproductive parameters were also analyzed in soil-grown plants irrigated and non-irrigated with the dual-strain bacteria. Two main treatment combinations were tested involving first the irrigation with the dual-strain followed by the inoculation with J2 from *M. javanica* three days later (treatment 1). The second treatment was firstly the inoculation with the *M. javanica* J2 followed by the irrigation with the dual-strain three days later (treatment 2). The reduction in the reproduction parameters in those tomato plants treated with the dual-strain respect to the untreated control were significant for all the combinations tested (**Figures 7A, B**). Hence the number of eggs at eight weeks post inoculation was reduced in 871 eggs per plant in treatment 1 and in 790 eggs per plant in treatment 2 respect to the untreated controls, what represents a percentage of reduction relative to the control of (75% and 68%, respectively; **Figure 7A**). Likewise, the index, eggs per roots gram weight was also remarkably affected with a significant reduction (p<0.05) of 500 and 550 eggs per root gram respect to the control in treatment 1 and 2, respectively. Therefore, all these data presented confirm the effectiveness of the dual-strain for the nematode control *in vitro* and soil. The inhibition of the reproduction seems to be effective regardless of the application of the bacteria before or after J2 inoculation (treatment 1 and 2; **Figure 7**). Respect to the growth parameters in tomato, the biomass of either the aerial parts or roots did not vary as a consequence of any of the treatments (**Figures S2C, D**).

**Figure 8:**
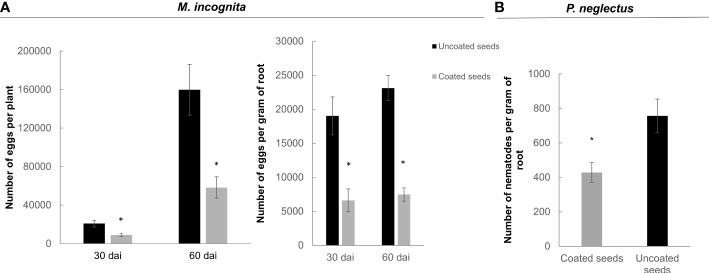
Nematode reproduction (*Meloidogyne* spp. and *Pratylenchus* spp.) in soybean plants from bacteria coated seeds. **(A)** Number of nematode eggs per plant (left) and nematode eggs/gram (right) of root of *Meloidogyne* spp. measured at 30 (one infection cycle) and 60 days (two infections cycles) after soybean inoculation with 5000 eggs of *M. incognita.* **(B)** Number of *Pratylenchus* spp. specimens measured 40 days after soybean sowing in nematode natural infested soil. The dual-strain combination of *B paralicheniformis* FMCH001 and *B subtilis* FMCH002 was applied by seed coating. Bars are mean ± standard error (n=9). Asterisk represents significant difference to non-treated control seeds (t-test; p < 0.05).

Furthermore, the ability of the dual-strain combination on inhibiting nematode reproduction was assessed in soybean for *Meloidogyne* spp. and *Pratylenchus* spp. The treatment applied by seed coating was efficient on controlling *M. incognita* population throughout the two life cycles analyzed: one life cycle (30 days), and two life cycles (60 days) after inoculation. Following the data presented in tomato (**Figure 7**), the number of eggs per root was sternly affected in the bacteria-coated soybean plants as compared to the control either 30- or 60-days post inoculation, with a reduction percentage of 56% and 63%, respectively (p<0.05; **Figure 8A**, left graph). When the number of eggs was normalized to the weight of the root, the differences were enhanced between the treatment and control, as the percentage of reduction was 57% at 30 dai and 64% at 60 dai (**Figure 8A**, right graph). Moreover, the seed coating with the dual-strain bacteria was efficient in reducing the population of another group of nematodes that are migratory, *Pratylenchus* by 44% (**Figure 8B**). The fresh weight of soybean roots did not vary in the treated plants as compared to the untreated, either with *Meloidogyne* spp. or *Pratylenchus* spp. (**Figures S2A, B**).

## Discussion

There is an increasing need to search for nematode control strategies compatible with IPM systems. In this respect, rhizobacteria within the *Bacillus* genus show multiple modes of action against PPNs that can act alone or in combination (reviewed in Miljaković et al., 2020; Ahmad et al., 2021). Thus, we evaluated the dual-strain combination of *B. paralicheniformis* FMCH001 and *B. subtilis* FMCH002 on PPNs control, with particular emphasis on RKNs. Firstly, we proved a direct effect on the pre-infective J2, resulting in high mortality rates, as well as reduction on egg hatching after the treatment with the dual-strain bacteria combination (**Figure 1**). Moreover, bacterial supernatant of each of the two *Bacillus* strains (FMCH001and FMCH002) caused also direct mortality after 48 hours incubation of *Meloidogyne* spp. J2 (**Figure 2**). These findings suggest that the secondary metabolites produced by each of the strains during vegetative cells growth, could be, at least partially, responsible for the direct nematicidal effect observed. It has been described that direct nematicidal activity can be due to secretion of secondary metabolites by *Bacillus* spp. (reviewed in Ahmad et al., 2021), one example is that diverse volatile compounds (ketones, acids, alkyls, sulfide, etc.) present in *B*. *subtilis* strain Bs-1, interfere with hatching and produce J2 mortality on *M. incognita* (Cao et al., 2019), or *Bacillus mycoides* strain R2 that produce the volatile styrene, very effective as a repellent and nematicidal against *M. incognita* (Luo et al., 2018).

It was also observed a significant reduction on juvenile attraction and penetration during the early stages of the tomato-*M. javanica* interaction in plants from bacteria treated roots as compared to untreated (**Figure 3**). Similar effects were also described with *Bacillus subtilis* strain Bs-1, which showed repellence of *M. incognita*, as well as effects on mortality and egg hatching (Cao et al., 2019). GC-MS assays detected that the major gas of the volatile compounds (VOCs) produced by this BS-1 strain was CO_2_ and other substances such as acetic acid, in this respect, the alterations of *Meloidogyne* spp. attraction by high concentrations of CO_2_ and variations on pH are well described (Pline and Dusenbery, 1987; Wang et al., 2009). Other examples are the treatment with bacterium *B. cereus* in soil that inhibited the penetration of *M. javanica* J2s (Oka et al., 1993) and caused repellence and *B. cereus* strain BCM2 with *M. incognita*, in tomato (Li et al., 2019). Therefore, it might be possible that the dual-strain combination of *B. paralicheniformis* FMCH001 and *B. subtilis* FMCH002 here studied, when colonizing the roots, can interfere with the recognition of plant exudates by the nematodes. Accordingly, it is known that rhizobacteria consume the exudates thereby truncating the nematode’s identification of root penetration points (reviewed in Lamovsek et al., 2013).

However, the dual-strain combination used, also remarkably reduced the number of galls per plant *in vitro* (**Figure 4**), as well as the reproduction parameters in soil, and this last effect was independent of the bacterial application mode, either before or after inoculation with the nematodes (**Figure 7**), hence, the versatility of those treatments seems a positive characteristic for IPM uses. Several studies observed a similar reduction in egg masses or root galling after *Meloidogyne* spp. infection in tomato, such as those treated with *Bacillus* spp. strains isolated from Iranian tomato fields (Ramezani-Moghaddam et al., 2014) or the *B. cereus* strain Jdm1 that decreased the root galling severity of *M. incognita* (43%) and promoted tomato growth performance (Xiao et al., 2018). Interestingly, the size of the galls, as well as GCs size was also reduced by more than 50% in the *in vitro* bacteria treated plants (**Figures 4**, **5**). These results indicate that the development of the feeding sites is somehow affected by the dual-strain combination of *B. paralicheniformis* FMCH001 and *B. subtilis* FMCH002 treatment. Similarly, *M. incognita* also produced few small galls in the tomato roots treated with *B. mycoides* R2 broth as compared to the untreated controls (Luo et al., 2018) and the treatment of tomato plants in greenhouses with *B. subtilis* showed fewer and smaller galls than those on water control plants (Cao et al., 2019). However, to our knowledge this is the first time that specifically, GCs size alterations have been reported after *Bacillus* treatments during *Meloidogyne* spp. infection. Additionally, we confirmed by confocal microscopy that the bacteria are present in the plant tissues and therefore can colonize the plant roots, including the galls, after the seeds coating treatment (**Figure 6**, **S4**). The effects observed in reducing the galls, but also the GCs growth could be directly due to altered plant responses to the presence of the bacteria, and/or to a direct or indirect effect of the bacteria on the nematodes at the J2, J3, J4 living stages, that could interfere with the proper GCs development. This last, could be due for example to alterations of the plant-nematode molecular crosstalk necessary for GCs formation and development. In this respect, bacterial metabolites are also able to produce a plant defense response that controls *Meloidogyne* spp. on tomato like hydrogen cyanide (Siddiqui et al., 2006). However, functional mechanisms for these described effects have not yet been proposed. Further research will elucidate the putative transduction pathways involved in those altered morphogenetic responses in galls and GCs after the bacterial treatment. Besides, the bacterial combination used was also effective in soybean, that showed a considerable reduction of the reproduction parameters after the dual-strain combination treatment, which was enhanced after two infection cycles (**Figure 8**). Moreover, the infection of nematodes of the genus *Pratylenchus* spp. was also considerably affected after the dual-strain treatment in soybean. This opens the possibility, that the dual-strain combination of *B. paralicheniformis* FMCH001 and *B. subtilis* FMCH002 could be effective against other groups of PPNs and in different crops.

In conclusion, our analysis confirmed the effectiveness of a combination of bacteria as a biological control method for *Meloidogyne* spp. in tomato in controlled conditions. We have evidence that direct mechanisms may act on the J2 pre-infective stage and on the eggs of *M. javanica*. Additionally, direct and/or indirect mechanisms promoted a decrease in the attraction and penetration of the J2 to the tomato roots after bacteria treatment, which indicates that the dual-strain combination could also interfere with root recognition. Furthermore, different mechanisms, still not well known, also interfered with galling and reproduction that were severely reduced. These altered reproduction parameters could be a consequence of the reduction in the GCs size, either through effects on the nematode (J3 and J4 life-stages), or on the plant development processes that lead to GCs formation (Escobar et al., 2015). Our findings are aligned with other studies showing different nematode control mechanisms by bacteria within the *Bacillus* genus. Some examples are *B. velezensis* strain Bv-25, that reduced nematode infection in laboratory assessments exhibiting also direct nematicidal against *M. incognita in vitro* (Tian et al., 2022). Likewise, inoculation of *B. megaterium* stimulated plant biomass, root length, shoot length, and nutrient uptake in tomato plants with declined RKNs population (El-Hadad et al., 2011). *M. incognita* were also controlled using an eco-friendly formulation from *B. subtilis* under laboratory and greenhouse conditions (Silva et al., 2017; Basyony and Abo-Zaid, 2018). Consequently, this study identified a potential bionematicide based on a dual-strain combination of *B. paralicheniformis* FMCH001 and *B. subtilis* FMCH002 that could be effective for better management of RKNs within an IPM scheme and described different modes of action of the dual-strain, including effects on GCs development. Considering the existing environmental challenges, it is essential to restrict the chemical nematicides available on the market as the regulations of all the states are demanding, yet many of them are already banned by many countries (Gad, 2014).

## Data availability statement

The raw data supporting the conclusions of this article will be made available by the authors, without undue reservation.

## Author contributions

CE, LM, DA, and FED-M conceived and designed the experiments. CE and LM provided resources and funding. CE coordinated the experiments. FED-M and AG-M performed most of the experiments. CE, FED-M, and DA wrote the manuscript. All authors contributed to the article and approved the submitted version.

## Supplementary material

Supplementary Figure 1 Effect of the dual-strain combination on *Meloidogyne javanica* J2 and eggs. **(A)** Number of living nematodes respect to the control. The J2 mortality was monitored for 96 hours. **(B)** *Meloidogyne javanica* alive or dead nematodes and non-hatched eggs were considered in the analysis and were monitored after ten days. 10mM and 5mM of magnesium sulfate were used as controls for the 1X and 1/2X formulations, respectively. Concentration used of dual-strain bacteria combination of *B. paralicheniformis* FMCH001 and *B. subtilis* FMCH002 were 3,20E+08 CFU/ml (1x bacteria) and 1,60E+08 CFU/ml (1/2x bacteria). Bars represent mean ± SE for 400 nematodes J2 or eggs tested per formulation and dual-strain concentrations or controls (10mM and 5mM of magnesium sulfate). *Asterisks represent significant differences with respect to control (t-test; P<0.05).

Supplementary Figure 2 Effect of dual-strain bacteria combination treatment of tomato and soybean on the plant fresh weight. Fresh weight of soybean infected with **(A)** *Meloidogyne incognita*, values were measured at 30 (one infection cycle) and 60 days (two infections cycles) after soybean inoculation with 5000 eggs*;* and **(B)** with *Pratylenchus*, values were measured at 40 days after soybean sowing in natural infested soil. Treatments were applied by seed coating with the dual-strain combination of *B. paralicheniformis* FMCH001 and *B. subtilis* FMCH002. Bars are mean ± stand error (n=9). **(C and D)** fresh weight of roots and aerial parts, respectively of tomato plants infected with *M. javanica*, values were measured 8 weeks after inoculation (one cycle of infection) with 400 J2s. Tomato treatments are detailed in materials and methods and correspond to the experiments in . Bars are mean ± stand error (n=24). Data were analyzed using *t-test* and no significance differences were encountered at p<0.05.

Supplementary Figure 3 Representative diagram of the attraction assay. Roots of tomato seedlings were immersed in a suspension of 1,0 x10^6 CFU/ml of the dual-strain combination of *B. paralicheniformis* FMCH001 and *B. subtilis* FMCH002 and placed in the plate with pluronic gel. After 24 hours, 600 J2 were distributed through five spots at the bottom of the plate. The number of juveniles that reached the roots was assessed after 24 and 48 hours, as well as the number of juveniles inside the roots and the number of galls formed on each root. Scale bar: 2cm.

Supplementary Figure 4 Sybrgreen staining of roots after bacteria treatment. **(A)** Non-treated roots; **(B)** Treated roots with a suspension of 1,0 x 10^6 CFU/ml of a dual-strain combination of *B. paralicheniformis* FMCH001 and *B. subtilis* FMCH002. Bacteria cells are indicated with the white arrows over the roots. Scale bar: 20 µm.
