# Supplementary material for: Biocontrol of Meloidogyne spp. in Solanum lycopersicum using a dual combination of Bacillus strains
Source: Front Plant Sci. 2023 Jan 4;13:1077062. doi: 10.3389/fpls.2022.1077062 (PMC9846617; doi:10.3389/fpls.2022.1077062)
Supplement: Supplementary Figure 1 — Effect of the dual-strain combination on Meloidogyne javanica J2 and eggs. (A) Number of living nematodes respect to the control. The J2 mortality was monitored for 96 hours. (B) Meloidogyne javanica alive or dead nematodes and non-hatched eggs were considered in the analysis and were monitored after ten days. 10mM and 5mM of magnesium sulfate were used as controls for the 1X and 1/2X formulations, respectively. Concentration used of dual-strain bacteria combination of B. paralicheniformis FMCH001 and B. subtilis FMCH002 were 3,20E+08 CFU/ml (1x bacteria) and 1,60E+08 CFU/ml (1/2x bacteria). Bars represent mean ± SE for 400 nematodes J2 or eggs tested per formulation and dual-strain concentrations or controls (10mM and 5mM of magnesium sulfate). *Asterisks represent significant differences with respect to control (t-test; P<0.05). [file Presentation_1.pdf]

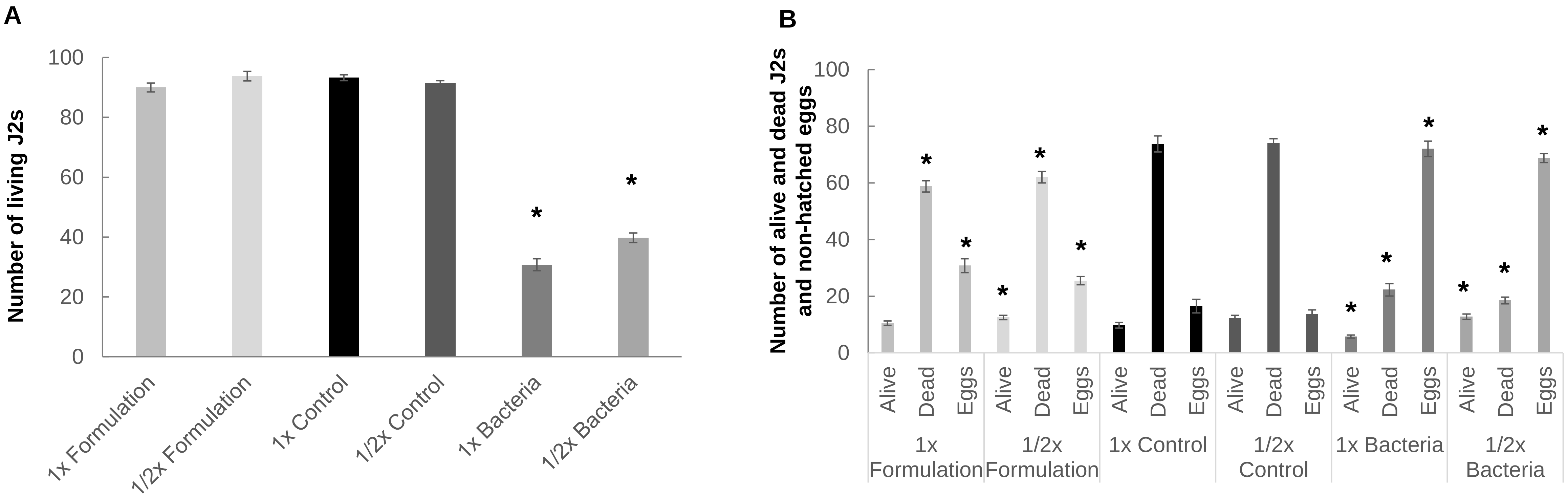

SUPPLEMENTARY FIGURE 1

Effect of the dual-strain combination on *Meloidogyne javanica* J2 and eggs. (A) Number of living nematodes respect to the control. The J2 mortality was monitored for 96 hours. (B) *Meloidogyne javanica* alive or dead nematodes and non-hatched eggs were considered in the analysis and were monitored after ten days. 10mM and 5mM of magnesium sulfate were used as controls for the 1X and 1/2X formulations, respectively. Concentration used of dual-strain bacteria combination of *B. paralicheniformis* FMCH001 and *B. subtilis* FMCH002 were 3,20E+08 CFU/ml (1x bacteria) and 1,60E+08 CFU/ml (1/2x bacteria). Bars represent mean ± SE for 400 nematodes J2 or eggs tested per formulation and dual-strain concentrations or controls (10mM and 5mM of magnesium sulfate). *Asterisks represent significant differences with respect to control (t-test;$P<0.05$).

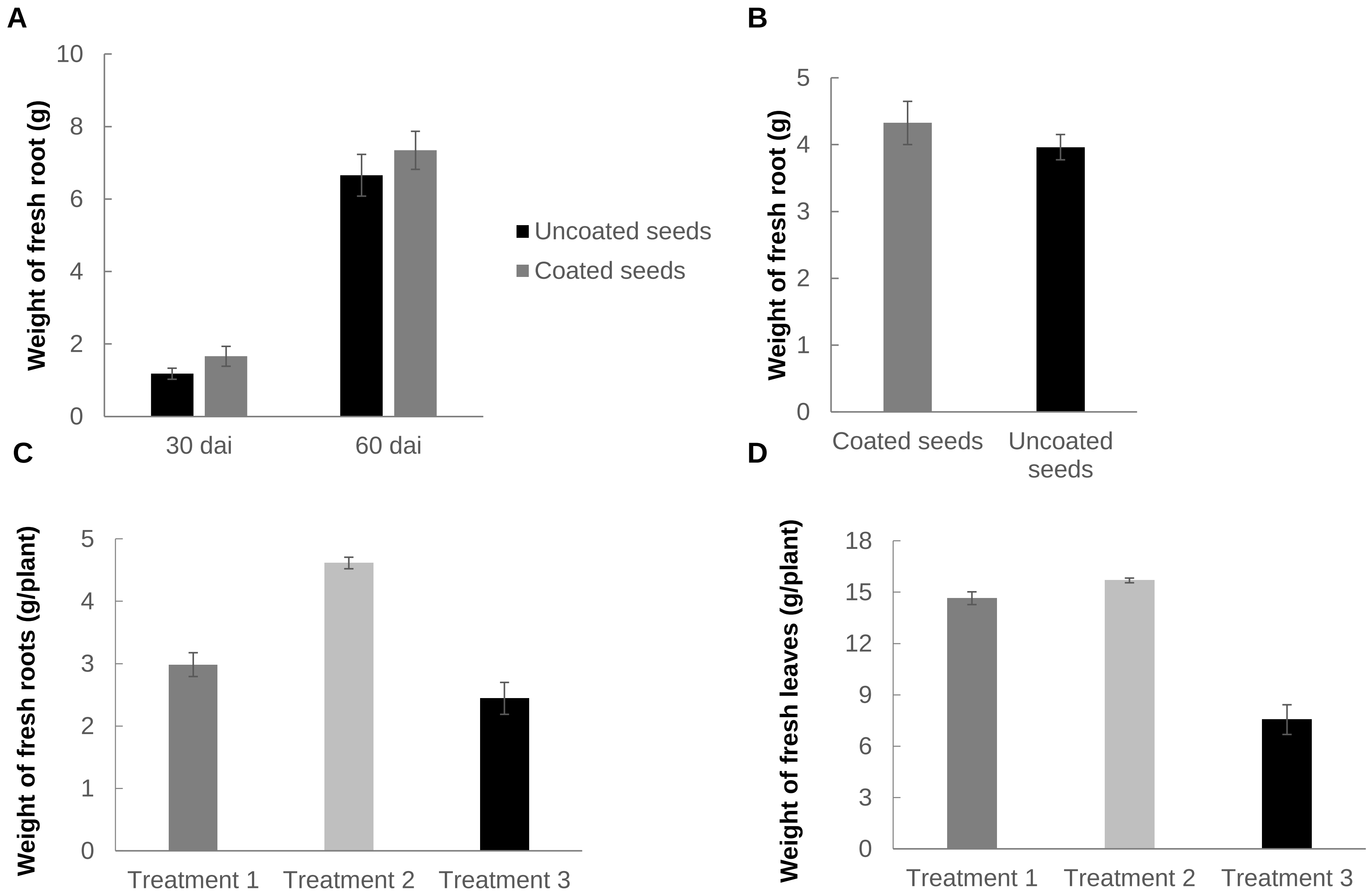

Fig. S2

SUPPLEMENTARY FIGURE 2

Effect of dual-strain bacteria combination treatment of tomato and soybean on the plant fresh weight. Fresh weight of soybean infectedwith (A) *Meloidogyne incognita*, values were measured at 30 (one infection cycle) and 60 days (two infections cycles) after soybean inoculation with 5000 eggs; and (B) with *Pratylenchus*, values were measured at 40 days after soybean sowing in natural infested soil. Treatments were applied by seed coating with the dual-strain combination of *B. paralicheniformis* FMCH001 and *B. subtilis* FMCH002. Bars are mean ± stand error (n=9). (C and D) fresh weight of roots and aerial parts, respectively of tomato plants infected with *M. javanica*, values were measured 8 weeks after inoculation (one cycle of infection) with 400 J2s. Tomato treatments are detailed in materials and methods and correspond to the experiments in Bars are mean ± stand error (n=24). Data were analyzed using t-test and no significance differences were encountered at $p<0.05$.

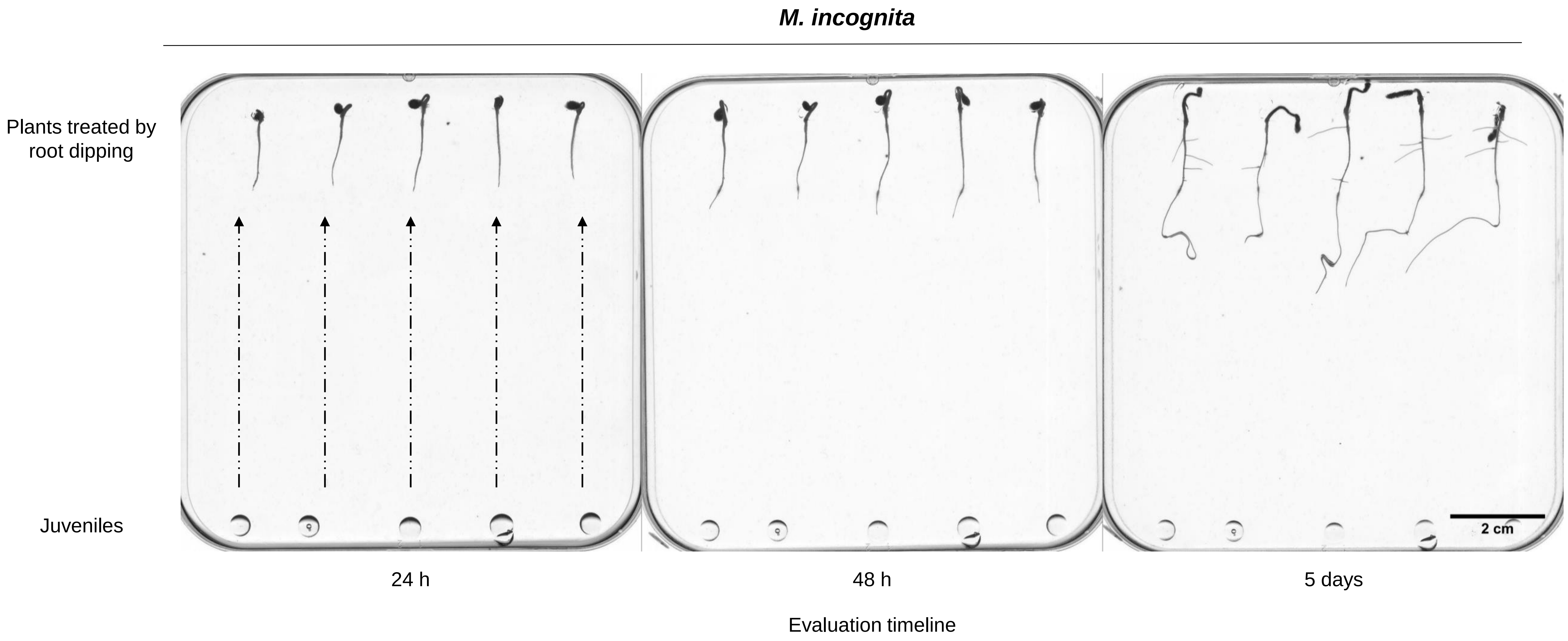

SUPPLEMENTARY FIGURE 3

Representative diagram of the attraction assay. Roots of tomato seedlings were immersed in a suspension of 1,0 x10^6 CFU/ml of the dual-strain combination of *B. paralicheniformis* FMCH001 and *B. subtilis* FMCH002 and placed in the plate with pluronic gel. After 24 hours, 600 J2 were distributed through five spots at the bottom of the plate. The number of juveniles that reached the roots was assessed after 24 and 48 hours, as well as the number of juveniles inside the roots and the number of galls formed on each root. Scale bar: 2cm.

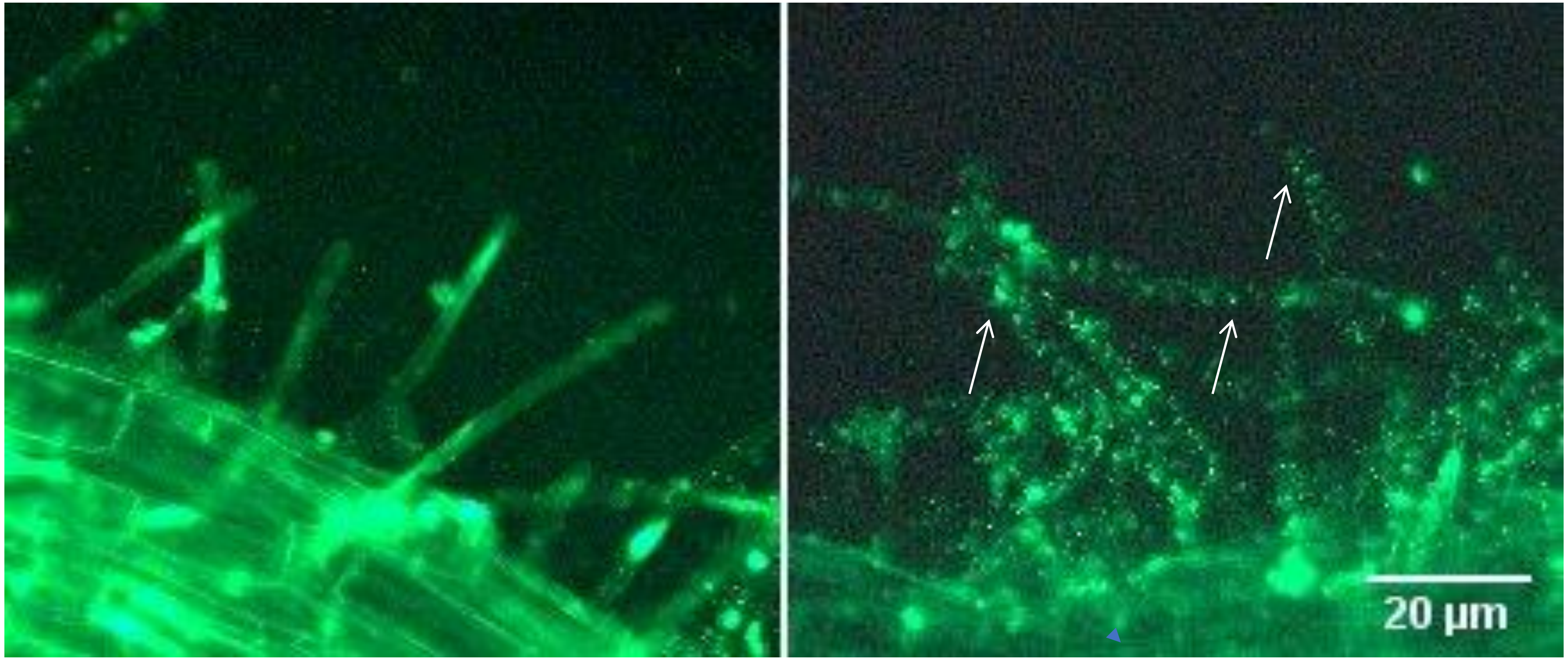

SUPPLEMENTARY FIGURE 4

Sybrgreen staining of roots after bacteria treatment. (A) Non-treated roots; (B) Treated roots with a suspension of 1,0 x 10 ^6 CFU/ml of a dual-strain combination of B. *paralicheniformis* FMCH001 and *B. subtilis* FMCH002. Bacteria cells are indicated with the white arrows over the roots. Scale bar: 20 µm.
